# The Afterglow Inventory (AGI): Validation of a new instrument for measuring subacute effects of classic serotonergic psychedelics

**DOI:** 10.1177/02698811251326937

**Published:** 2025-03-31

**Authors:** Tomislav Majić, Timo Torsten Schmidt, Anna Gröticke, Peter Gasser, William A Richards, Thomas G Riemer, Ricarda Evens

**Affiliations:** 1 Department of Psychiatry and Psychotherapy, Berlin Institute of Health, Charité Universitätsmedizin Berlin, Humboldt-Universität zu Berlin, Berlin, Germany; 2 Neurocomputation and Neuroimaging Unit, Department of Education and Psychology, Freie Universität Berlin, Berlin, Germany; 3 Medical Office for Psychiatry and Psychotherapy, Solothurn, Switzerland; 4 The Bill Richards Center for Healing, Sunstone Therapies, Rockville, MD, USA; 5 Charité—Universitätsmedizin Berlin, Humboldt‑Universität zu Berlin, and Berlin Institute of Health, Institute of Clinical Pharmacology and Toxicology, Berlin, Germany; 6 Department of Psychology, Humboldt-Universität zu Berlin, Berlin, Germany

**Keywords:** Psychedelic, afterglow, subacute, questionnaire, assessment

## Abstract

**Background:**

Classic psychedelics such as psilocybin and lysergic acid diethylamide are anecdotally associated with the phenomenon of “psychedelic afterglow,” a set of predominantly pleasant, temporary psychological effects reported after the acute effects have subsided. Since post-acute effects are crucial for the therapeutic use of psychedelics, an instrument to systematically assess subacute “afterglow” effects is needed.

**Aims:**

To create and validate a questionnaire to quantify the subacute “afterglow” effects of psychedelics.

**Methods:**

An international online survey was conducted in English and German. Participants who had consumed a psychedelic (*N* = 1323) or another non-psychedelic substance (control group, *N* = 157) within the past 4 weeks were included. An initial list of 97 items was progressively reduced to 24 items.

**Results:**

A 5-factor structure best fit the data and showed high internal consistency. The factors included (1) vitality, (2) transpersonal aspects, (3) inspiration/creativity, (4) interpersonal relationships, and (5) relationship to nature. The final 24-item version of the Afterglow Inventory (AGI) effectively differentiated between the psychedelic group and the control group. The overall AGI score positively correlated with the intensity (*r* = 0.165; *p* < 0.001) and positive valence (*r* = 0.251; *p* < 0.001) of the acute psychedelic effects.

**Conclusions:**

The AGI is a novel scale for quantifying positive subacute (“afterglow”) effects of psychedelics. The use of the AGI could lead to a better understanding of the interplay between acute, subacute, and long-term effects of psychedelics. Insights could also be gained into how different substances, dosages, and extra-pharmacological factors, such as psychotherapy, might influence outcomes.

## Introduction

In recent decades, classic serotonergic psychedelics (“psychedelics”), such as lysergic acid diethylamide (LSD), psilocybin, and the Amazonian concoction ayahuasca, have been rediscovered as tools for investigating human consciousness. In addition, psychedelics have been proposed for treating certain mental health conditions (Reiff et al., 2020), such as treatment-resistant depression (Carhart-Harris et al., 2021; Goodwin et al., 2022), substance-use disorders (Bogenschutz et al., 2022), and depression and anxiety in patients with life-threatening illness (Reiche et al., 2018).

Despite their structural variety, the psychoactive effects of substances from the group of classic psychedelics exhibit strong phenomenological overlaps (Hirschfeld and Schmidt, 2021; Hirschfeld et al., 2023; Prugger et al., 2022). For instance, findings from comparison studies assessing altered states of consciousness (ASCs) fail to discriminate between LSD, psilocybin, and mescaline (Ley et al., 2023).

Psychedelics are unique in the temporal dynamics of their psychoactive effects (Majić et al., 2015). Unlike most other psychoactive substances, the effects of psychedelics can outlast their acute effects, exhibiting acute, subacute, and long-term effects (Studerus et al., 2011). Notably, even a single dosage of psilocybin might occasion post-acute effects that may last up to more than 12 months (Griffiths et al., 2008). Various models have been suggested to explain the mechanisms of these post-acute effects. Converging evidence from recent studies indicates that psychedelics promote neuroplasticity, possibly by activating intracellular 5HT2a receptors via AMPA receptors, TrkB, and mTOR signaling (Vargas et al., 2023), possibly representing neural correlates of subacute effects. Initial studies suggest that ayahuasca might differentially affect network connectivity within the salience network and the default mode network 1 day after administration (Pasquini et al., 2020). Furthermore, increased global integration has been reported in patients suffering from major depression 1 day and 3 weeks after using psilocybin, suggesting improved network interconnectivity and flexibility (Daws et al., 2022).

Per definition, *post-acute* effects encompass all psychological effects following the ingestion of psychedelics observed once the *acute* effects have worn off (Goldberg et al., 2020). Specifically, post-acute effects include *subacute* effects embracing a timeframe of between 1 day and 1 month after using a psychedelic, as well as *long-term* effects, persisting for months or even years (Studerus et al., 2011). However, the distinction between subacute and long-term effects lacks clarity across studies. Importantly, the subacute window is often associated with a variety of predominantly beneficial psychological effects (for a review, see Evens et al., 2023). These include, among others, improvement of general indicators of psychopathology (Jiménez-Garrido et al., 2020), elevated mood (Schmid and Liechti, 2018), well-being (Haijen et al., 2018), quality of life (Anderson et al., 2020), increased mindfulness (Soler et al., 2016), and cognitive flexibility (Basedow et al., 2021).

These positive psychological phenomena have anecdotally been labeled as “psychedelic afterglow” or “carry-over effect” by various authors (e.g., Albaugh and Anderson, 1974; Halpern, 1996; Murphy-Beiner and Soar, 2020; Pahnke et al., 1970; Sampedro et al., 2017). Descriptions characterize these phenomena not only by elevated mood and increased energy but also by interpersonal and spiritual openness (Pahnke, 1967). It has been suggested that afterglow effects might enhance the responsiveness for psychotherapeutic interventions during the subacute window (Murphy-Beiner and Soar, 2020).

The term “psychedelic afterglow” was first described by Walter Pahnke and colleagues (1970), associated with observations from clinical research with LSD:

If a psychedelic peak experience has been achieved and stabilized during the session, a clinical picture which we have termed the *psychedelic afterglow* can be observed in the days after the session. Mood is elevated and energetic; there is a relative freedom from concerns of the past and from guilt and anxiety, and the disposition and capacity to enter into close interpersonal relationships is enhanced. These psychedelic feelings generally persist for from 2 weeks to a month and then gradually fade into vivid memories that hopefully will still influence attitude and behavior. During this immediate post drug period, there is a unique opportunity for effective psychotherapeutic work on strained family or other interpersonal relationships. (Pahnke et al., 1970, p. 1858)

Another study investigating the treatment of terminally ill patients with LSD emphasized spiritual and existential dimensions as core aspects of these phenomena:

radiant and positive feeling of well-being that often connotes a real change in values, an increase in spirituality, a decrease in meaningless goals, less emphasis on material things, a feeling of being more at home in life and a greater appreciation of life’s possibilities. (Kurland, 1985a, p. 283)

In summary, the “psychedelic afterglow” has been conceptualized as a predominantly positive, transient phenomenon following the acute effects of classic psychedelics, including improvements in mood, interpersonal relationships, and increased openness to spiritual and existential aspects of life. Importantly, although many users report psychedelic afterglows, they are not universally experienced.

Afterglow phenomena seem to be unique to psychedelics, as no such effects have been reported for most other psychoactive substances. Conversely, other substance groups have been linked to unpleasant hangover effects emerging once the acute effects have worn off, such as alcohol, stimulants, or MDMA, which have been associated with “mid-week blues.”

While several instruments assessing the *acute* effects of psychedelics are available (for an overview, see Prugger et al., 2022), to date we are not aware of any tool specifically assessing subacute effects or “psychedelic afterglow” phenomena. Therefore, this work aimed to design and validate a tool for the assessment of “afterglow phenomena,” providing a valuable resource in assessing and comparing the intensity and duration of these effects.

## Methods

### Item generation

To capture the “psychedelic afterglow,” an item pool was generated based on previous descriptions of the phenomenon (e.g., Albaugh and Anderson, 1974; Kurland, 1985b; Pahnke, 1969; Pahnke et al., 1970), and by identifying possibly related items of other scales (e.g., the Persisting Effects Questionnaire (Griffiths et al., 2006), the Watts Connectedness Scale (Watts et al., 2022), the Nature Relatedness Scale (Nisbet et al., 2009), the Mystical Experience Questionnaire (MacLean et al., 2012), the Adult Self-Transcendence Inventory (Levenson et al., 2005), and the Multidimensional Assessment of Interoceptive Awareness (Mehling et al., 2012)). Duplicates were removed, all items were brought into uniform wording and were sorted by categories which served as the basis for the formulation of further items in an iterative process. All items were generated simultaneously in English and German language to ensure maximum comparability between both versions. The initial item list was presented to four expert advisors (William A. Richards, Rick Strassman, Ulrich Ott, and Peter Gasser) who rated the relevance of each item and provided feedback on item formulation. The item list was refined and reduced to an item pool of 97 items that underwent a language review by a bilingual German and English native speaker. The resulting items were taken into the validation process described below and are presented in Supplemental Table S1.

Two different sets of instructions were developed to create a “change version” and a “state version” of the questionnaire that would allow to use it either (1) to assess the *change* from a previous state (“Please judge to what extent the statements apply to your experience during the days following your last consumption of the substance as compared to your normal/everyday experience.”) or (2) to assess the current *state* of a person (“Please rate to what extent the statements apply to your experience at this point in time (today).”). Of note, items of both versions of the questionnaire are identical, whereas only instructions and response options differ. In the online survey described here, we used the change version of the questionnaire. In this version, a visual analog scale (0–100) with the anchors zero defined as “NO, not more than usual” and a hundred defined as “YES, much more than usual” was chosen as the response format.

### Study procedure

Between February 21, 2020, and March 4, 2021, English- and German-speaking volunteers were invited to complete an anonymous cross-sectional online survey called “The Afterglow Survey” (AGS). Participants were recruited via social media posts and e-mail announcements. Interested participants could inform themselves about the goals of the survey on a project landing page. From there, participants were redirected to the survey that was hosted on the secure online platform “SoSci Survey” (Leiner, 2019a), where they gave informed consent and were asked to fill out the questionnaire described below.

To be admitted to the survey, participants had to (1) indicate a minimum age of 18 years, (2) be fluent in German or English language, and (3) have used at least one substance in the last 4 weeks that was either (3a) a substance from the group of psychedelics (psychedelic group): LSD, lysergic acid amide (LSA), psilocybin, *N,N*-dimethyltryptamine (DMT), 5-methoxy-DMT (5-MeO-DMT), ayahuasca, mescaline, 2,5-dimethoxy-4(n)-propylphenethylamine (2C-B), or other classic psychedelics, or (3b) one of the following substances (non-psychedelic control group): amphetamine, methamphetamine, cocaine, or opioids. Those were selected as control substances as they do not act primarily via serotonergic pathways and in usual dosages they are not known for inducing psychedelic effects. Participants did not receive compensation for participation. The study protocol was approved by the Charité Ethics Committee (EA2/185/17).

### Survey structure

If participants met the inclusion criteria, they were asked the following details about the index substance they had indicated to have used in the last 4 weeks: dosage, route of administration, the subjective intensity of the acute effects, the subjective valence of the acute effects, and retrospective evaluation of the substance-related experiences (visual analog scale from “negative” to “positive”). In addition, the frequency of previous experiences with the substance and frequency of previous psychedelic experiences in general were assessed. Participants were then asked to respond to the initial set of 97 Afterglow Inventory (AGI) items (see section item generation). Furthermore, five control items on negative subacute effects were included to assess the specificity of subacute afterglow effects (“I feel angry,” “I am depressed,” “I feel anxious,” “I have feelings of inner tension,” “I easily become irritable”). Participants were also asked to indicate the overall duration of subacute effects.

At the end of the survey, demographical aspects were assessed including sex, age, country of residence, occupational status, and level of education. Furthermore, participants were asked to report about other previous experiences with psychedelic and non-psychedelic drugs.

### Data analysis

Data sets were excluded before data analysis if they showed one of the following indicators of poor data quality: (a) termination of the survey before completion of the 97 items of the initial AGI or (b) completion with more than 10% missing values, (c) relative speed index on SoSci Survey’s TIME_RSI index was above the recommended cutoff of 2.0 (Leiner, 2019b), or (d) free-text comments indicating that the survey was not filled out accurately.

Characteristics of participants and reported psychedelic experiences were described and compared between the English and German samples and the psychedelic and non-psychedelic samples. Exploratory factor analysis (EFA) was used to explore the underlying factor structure of the AGI and to select the final items. The analyses were performed with the full English sample of the psychedelic group. Principal axis factoring (based on a correlation matrix) with an oblique rotation method (Promax) was applied. The Kaiser criterion, parallel analysis (Horn, 1965; O’Connor, 2000), and visual inspection of the data were used to define the maximum number of factors retained. Items were selected for the final AGI if loadings were ⩾0.4 and if items did not load highly on different factors. If multiple items remained, further criteria were considered (e.g., standard psychometric properties, such as item selectivity and item difficulty). Furthermore, items were kept if the item felt important to capture the full construct of afterglow (face validity).

The factor structure of the reduced AGI was replicated in the German sample using confirmatory factor analysis (CFA; using the software Mplus 8). Following recommendations, multiple fit indices were calculated to assess goodness-of-fit (Brown, 2015; Hu and Bentler, 1999), including the root-mean-square error of approximation (RMSEA), the confirmatory fit index (CFI), and the standardized root mean square residual (SRMR). The reliability of the final item selection was calculated using Cronbach’s alpha.

To assess construct validity, a repeated measures analysis of variance (ANOVA) with the within-group factor “scale” (mean score of the final AGI selection vs. mean score of control items) and the between-group factor “group” (psychedelic vs. non-psychedelic groups) was conducted. We expected a significant interaction of scale and group, and the following significant post hoc tests (Bonferroni): (1) Higher AGI mean scores in the psychedelic compared to the non-psychedelic group (2) Lower mean scores of control items compared to AGI-only in the psychedelic group.

Furthermore, the association of the afterglow with features of the acute drug experience was explored. Correlations between AGI mean score and the subjective strength of effects (“How strong was the acute effect of the substance?” from 0 “no effect at all” to 100 “very strong effect”) and retrospective evaluation of the acute experiences (“How do you evaluate the experiences you had by taking the substance in retrospect?” from 0 “negative” to 100 “positive”) were performed using Pearson’s product–moment correlation coefficient. Lastly, differences in AGI mean scores between psychedelic substances were descriptively explored.

If not indicated otherwise, analyses were performed using SPSS (PASW Statistics Version 18.0, Chicago: SPSS Inc.)

## Results

### Participants

Of 4583 volunteers who started the survey, 3103 participants were excluded for the following reasons: inclusion criteria not fulfilled (*n* = 174), relative speed index exceeded the recommended cutoff of ⩾2 (*n* = 173), AGI items were not completed (*n* = 2729), indicated in the comments that the participant was just reading (*n* = 1), and number of missing AGI items exceeding 10% (*n* = 26). Of the remaining 1480 participants, 1323 reported experiences with a psychedelic substance (psychedelic group, see Table 2), 157 participants reported experiences with amphetamine (*n* = 61), methamphetamine (*n* = 12), cocaine (*n* = 57), or opioids (*n* = 27) (control group). This left a sample of 1323 psychedelic users stemming from 61 different countries.

Characteristics of the psychedelic group are presented in Table 1. The English-speaking sample (74.1%) reported 55 different countries of residence, the most frequently being the United States of America (46.1%), Canada (7.8%), and England (7.4%). The German-speaking sample (25.9%) reported six different countries of residence, the most frequent being Germany (87.4%), Switzerland (6.1%), and Austria (3.5%). Significant differences between the English- and German-speaking samples were observed in moderate effect sizes (⩾0.2) for the variables “education” and “experiences with the substance 2C-B” (see Table 1).

**Table 1.** Sample characteristics of the psychedelic group.

Sample characteristic	Total sample		English sample		German sample		*df*	*t* or χ²	*p*	Effect size^a^
	*N*	%	*N*	%	*N*	%				
	1323	100%	981	74.1%	342	25.9%				
Age (SD)	29.84	(10.47)	29.68	(10.94)	30.28	(9.03)	705	0.99	0.324	0.058
Sex							2	1.95	0.376	0.039
Female	305	23.1	233	23.8	72	21.1				
Male	942	71.2	685	69.8	257	75.1				
Other	28	2.1	22	2.2	6	1.8				
Education							2	75.77	<0.001	0.243
No degree	131	9.9	129	13.1	2	0.6				
Secondary school	550	41.6	350	35.7	200	58.5				
University	600	45.4	467	47.6	133	38.9				
Work situation							5	19.96	0.001	0.125
Student/apprentice	406	30.7	290	29.6	116	33.9				
Employed	517	39.1	395	40.3	122	35.7				
Self-employed/freelancer	156	11.8	97	9.9	59	17.3				
Unemployed	108	8.2	87	8.9	21	6.1				
Retired	28	2.1	24	2.4	4	1.2				
Other	66	5.0	53	5.4	13	3.8				
Experiences with psychedelics										
LSD	1108	83.7	803	81.9	305	89.2	1	7.23	0.007	0.075
LSA	227	17.2	138	14.1	89	26.0	1	24.10	<0.001	0.136
Psilocybin	1050	79.4	771	78.6	279	81.6	1	0.45	0.505	0.019
DMT	482	36.4	327	33.3	155	45.3	1	14.11	<0.001	0.104
5-MeO-DMT	119	9.0	82	8.4	37	10.8	1	1.66	0.197	0.036
Ayahuasca	173	13.1	119	12.1	54	15.8	1	2.60	0.107	0.045
Mescaline	210	15.9	139	14.2	71	20.8	1	7.64	0.006	0.077
2C-B	394	29.8	230	23.4	164	48.0	1	70.37	<0.001	0.233
Other psychedelics	521	39.4	354	23.4	167	48.8	1	15.47	<0.001	0.109
Number of previous psychedelic experiences							5	40.35	<0.001	0.176
1–5 days	310	23.4	265	27.0	45	13.2				
6–20 days	431	32.6	320	32.6	111	32.5				
21–50 days	280	21.2	184	18.8	96	28.1				
51–99 days	116	8.8	80	8.2	36	10.5				
100–200 days	91	6.9	56	5.7	35	10.2				
>200 days	78	5.9	60	6.1	18	5.3				
Previous experiences with other substances										
Alcohol	1221	92.3	909	92.7	312	91.2	1	4.66	0.031	0.060
Nicotine	1027	77.6	757	77.2	270	78.9	1	0.02	0.882	0.004
Cannabinoids	1224	92.5	905	92.3	319	93.3	1	0.22	0.642	0.013
MDMA	902	68.2	632	64.4	270	78.9	1	21.70	<0.001	0.129
Amphetamine	628	47.5	416	42.4	212	62.0	1	36.07	<0.001	0.167
Methamphetamine	168	12.7	128	13.0	40	11.7	1	0.57	0.450	0.021
Cocaine	649	49.1	464	47.3	185	54.1	1	3.59	0.058	0.053
Ketamine	507	38.3	326	33.2	181	52.9	1	38.93	<0.001	0.173
Opioids	464	35.1	341	34.8	123	36.0	1	0.04	0.849	0.005
Sedatives	534	40.4	431	43.9	103	30.1	1	22.45	<0.001	0.132
Previous experiences with antidepressants										
SSRIs	319	24.1	277	28.2	42	12.3	1	37.19	<0.001	0.170
SNRIs	110	8.3	94	9.6	16	4.7	1	8.44	0.004	0.081
MAO inhibitors	120	9.1	76	7.7	44	12.9	1	7.50	0.006	0.076

5-MeO-DMT: 5-methoxy-DMT; DMT: *N,N*-dimethyltryptamine; LSA: lysergic acid amide; LSD: lysergic acid diethylamide; MDMA: 3,4, methylenedioxymethamphetamine; SSRI: selective serotonin reuptake inhibitor; SNRI: serotonin and norepinephrine reuptake inhibitor.

a Cohen’s *d* for continuous data, Cramer’s *V* for nominal data.

Participants exhibited overall high levels of education, with less than 10% being currently unemployed. Comparison of the psychedelic group and the non-psychedelic group revealed significant differences in age, sex, education, and work situation, but the effect sizes were small, indicating minimal influence on the results.

Psychedelic users reported predominantly low to moderate previous experiences with psychedelics, with almost 25% reporting lifetime use of psychedelics between 1 and 5 days, and 32.6% reporting use on 6–20 days. Consumption patterns were comparable between the English and the German samples. Regarding lifetime use, 2C-B was the only substance showing a significant difference of moderate effect size between the English and the German samples. 2C-B use was reported by almost half of the participants from the German sample, whereas less than one in four English speakers indicated experiences with this substance.

### Characteristics of reported psychedelic index experiences

The “psychedelic index experience” was defined as an experience with a psychoactive (psychedelic or control) that had taken place no more than 4 weeks prior to questionnaire completion. Participants from the psychedelic group might have also used one of the control substances during that frame, whereas participants were assigned to the control group only if they had not used psychedelics.

In our sample, more than 75% of participants reported having used LSD or psilocybin for the index experience, and more than 80% reported using moderate or high dosages. By contrast, the other psychedelics exhibited relatively small sample sizes in our sample (see Table 6).

Rather unpleasant index experiences were reported by less than 2% of participants, whereas 30% reported experiences that embraced both pleasant and unpleasant elements.

Regarding the non-psychedelic control group, more than half of the reported index experiences were with amphetamine-type stimulants (i.e., amphetamine or methamphetamine). Taken together, the sample appears to display no relevant biases and is suited for the factor analyses.

Characteristics of the psychedelic index experience reported by the psychedelic group are presented in Table 2. Several differences between the experiences of the English and German samples were observed. The only difference with a moderate effect size was the subjective intensity of the acute experience with the English-speaking sample reporting a higher intensity.

**Table 2.** Characteristics of psychedelic index experience reported by the psychedelic group.

Psychedelic index experience	Total sample		English sample		German sample		*df*	*t* or χ²	*p*	Effect size^a^
	*N*	%	*N*	%	*N*	%				
	1323	100%	981	74.1%	342	25.9%				
Psychedelic used							7	37.88	<0.001	0.169
LSD or derivative	607	45.9	429	43.7	178	52				
LSA	9	0.7	3	0.3	6	1.8				
Psilocybin	418	31.6	339	34.6	79	23.1				
DMT	108	8.1	68	6.9	39	11.4				
5-MeO-DMT	31	2.3	27	2.8	4	1.2				
Ayahuasca	53	4.0	46	4.7	7	2.0				
Mescaline	13	1.0	11	1.1	2	0.6				
2C-B	85	6.4	58	5.9	27	7.9				
Date of use							4	22.64	<0.001	0.131
Today	56	4.2	46	4.7	10	2.9				
1–7 days ago	607	45.9	475	48.4	132	38.6				
8–14 days ago	284	21.5	211	21.5	73	21.3				
15–21 days ago	149	11.3	106	10.8	43	12.6				
22–28 days ago	227	17.2	143	14.6	84	24.6				
Subjective dosage strength							2	2.34	0.311	0.042
Low	308	23.3	227	23.1	81	23.7				
Medium	778	58.8	569	58.0	209	61.1				
High	237	17.9	185	18.9	52	15.2				
Subjective intensity of acute effects 0–100 (SD)^b^	62.14	(24.60)	63.55	(24.74)	58.07	(23.77)	1318	−3.56	<0.001	−0.224
Rating of acute effects							4	10.11	0.039	0.087
Rather pleasant	887	67.0	635	64.7	252	73.7				
Rather unpleasant	24	1.8	19	1.9	5	1.5				
Both pleasant and unpleasant	377	28.5	297	30.3	80	23.4				
Neither pleasant nor unpleasant	30	2.3	26	2.7	4	1.2				
I don’t know	5	0.4	4	0.4	1	0.3				
Retrospect evaluation of experiences 0–100 (SD)^c^	88.54	(16.37)	88.56	(16.46)	88.48	(16.11)	1321	−0.08	0.935	−0.005
Previous experiences with a specific substance							5	12.32	0.031	0.097
1–5 days	491	37.1	381	38.8	110	32.2				
6–20 days	442	33.4	323	32.9	119	34.8				
21–50 days	194	14.7	140	14.3	54	15.8				
51–100 days	79	6.0	51	5.2	28	8.2				
101–200 days	52	3.9	32	3.3	20	5.8				
>200 days	48	3.6	38	3.8	10	2.9				
Duration of aftereffects							6	19.13	0.004	0.121
Not at all	18	1.4	16	1.6	2	0.6				
A few hours	97	7.3	77	7.8	20	5.8				
1–3 days	324	24.5	246	25.1	78	22.8				
4–7 days	224	16.9	155	15.8	69	20.2				
8–14 days	124	9.4	102	10.4	22	6.4				
>14 days	94	7.1	76	7.7	18	5.3				
Until today	425	32.1	293	29.9	132	38.6				
Concomitant drug use										
Any	650	49.1	494	51.7	156	46.0	1	3.20	0.074	0.050
Alcohol	162	12.2	126	12.8	36	10.5	1	1.51	0.219	0.034
Nicotine	331	25.0	243	24.8	88	25.7	1	0.04	0.852	0.005
Cannabinoids	412	31.1	323	32.9	89	26.0	1	6.60	0.010	0.071
MDMA	56	4.2	41	4.2	15	4.4	1	0.01	0.916	0.003
Amphetamine	30	2.3	22	2.2	8	2.3	1	0.00	0.953	0.002
Methamphetamine	5	0.4	5	0.5	3	0.9	1	1.72	0.182	0.037
Cocaine	19	1.4	15	1.5	4	1.2	1	0.26	0.607	0.014
Ketamine	41	3.1	20	2.0	21	6.1	1	13.71	<0.001	0.103
Opioids	16	1.2	11	1.1	5	1.5	1	0.21	0.644	0.013
Sedatives	19	1.4	18	1.8	1	0.3	1	4.37	0.037	0.058
SSRIs	23	1.7	23	2.3	3	0.9	1	8.31	0.004	0.080
SNRIs	6	0.5	6	0.6	3	0.9	1	2.14	0.144	0.041
MAO inhibitors	19	1.4	14	1.4	5	1.5	1	0.00	0.991	0.000

5-MeO-DMT: 5-methoxy-DMT; DMT: *N,N*-dimethyltryptamine; LSA: lysergic acid amide; LSD: lysergic acid diethylamide; SD: standard deviation.

a Cohen’s d for continuous data, Cramer’s V for nominal data

b “How strong was the acute effect of the substance?.”

c “How do you evaluate the experiences you had by taking the substance in retrospect?” from “0” (negative) to “100” (positive).

### Item selection and model replication

All 97 items of the initial item pool were entered into an EFA that was performed using the data of the English psychedelic group (*n* = 981). Before analysis, the factorability of matrices was evaluated and all requirements were satisfied: Kaiser–Meyer–Olkin measure of sampling adequacy was above 0.7 (KMO = 0.987) and Bartlett’s test of sphericity was significant (χ^2^(4656) = 80,194.47, *p* < 0.0001), an inspection of the anti-image correlation matrix yielded that all diagonals were >0.5. The inspection of the correlation matrix generally revealed sufficient correlations between the variables; however, three items showed correlations of less than 0.30 with most other variables (“I feel desire for sex,” “My mood changes a lot,” “I need little sleep”) and were excluded in the first round of item reduction.

The Kaiser criterion indicated a factor solution with the extraction of a maximum number of 10 factors, parallel analysis indicated a factor solution with a maximum of 15 factors. Visual inspection of the screen plot yielded a strong first factor and a level-off after five factors. After the reduction of items based on the prespecified criteria in four consecutive steps, a five-factor solution with 24 items yielded the best fit for the data, accounting for 65% of the variance. Communalities of the 24 variables ranged from 43% to 81%. Factor loadings of the final five-factor solution of the EFA are presented in Table 3. Printable versions of the English and German questionnaires are in Supplemental Appendices A and B.

**Table 3.** Factor loading of exploratory factor analysis in the final selection of 24 items.

Items			Factors		
	1	2	3	4	5
Item 14: I am in a good mood.	**0.952**	−0.241	0.002	0.038	−0.025
Item 08: I have enthusiasm for life in general.	**0.781**	0.052	−0.097	0.093	0.030
Item 12: I experience inner peace.	**0.673**	0.046	0.125	−0.042	0.037
Item 02: I am thankful for my life.	**0.666**	0.084	−0.124	0.002	0.092
Item 01: I feel comfortable in my body.	**0.640**	−0.021	−0.018	0.111	−0.056
Item 07: I feel connected to the beauty of life.	**0.582**	0.248	−0.053	−0.078	0.217
Item 11: I feel cleansed.	**0.557**	0.135	0.165	−0.039	−0.070
Item 16: I have deep feelings of joy.	**0.543**	−0.067	0.263	0.137	−0.014
Item 06: I feel that everything is interconnected.	0.000	**0.883**	0.008	0.020	−0.023
Item 24: I feel that all is one.	0.004	**0.820**	−0.016	0.071	−0.033
Item 05: I am thinking about spiritual or religious topics.	−0.189	**0.677**	0.026	0.052	0.088
Item 09: I feel connected to deeper aspects of myself.	0.255	**0.580**	0.107	−0.029	−0.047
Item 20: I feel creative.	−0.009	−0.059	**0.877**	−0.039	0.015
Item 23: My thinking is imaginative.	−0.121	0.092	**0.752**	0.051	0.049
Item 15: I feel inspired.	0.212	0.055	**0.618**	−0.004	−0.066
Item 10: I am discovering lost aspects of my life, such as feelings and desires.	0.050	0.225	**0.480**	0.011	0.020
Item 13: I feel emotionally connected to other people.	0.046	0.061	−0.003	**0.824**	−0.004
Item 17: I feel compassion toward other people.	0.114	0.073	−0.102	**0.815**	0.004
Item 04: I feel love toward other people.	0.046	0.090	0.038	**0.731**	0.038
Item 19: I am willing to engage in close relationships.	0.075	−0.114	0.277	**0.499**	0.066
Item 22: I notice nature around me.	−0.087	−0.074	0.059	0.062	**0.931**
Item 03: I enjoy being in contact with nature.	0.128	0.027	−0.110	−0.005	**0.766**
Item 18: I feel connected to nature.	0.010	0.158	0.075	−0.026	**0.729**
Item 21: I perceive beauty even in small details (e.g., a human voice, a flower, a work of art).	0.177	0.002	0.238	0.038	**0.429**

Sample: *n* = 981 participants of the English psychedelic sample; Extraction method: principal axis factoring (based on correlation matrix) with an oblique rotation method (Promax). Loadings larger than 0.40 are in bold.

Factor 1 explained 54% of the variance and comprised items that express a distinct sense of *vitality*, including intra-subjective aspects of well-being and gratefulness. Factor 2 explained a further 4% of the variance and included items that describe the engagement with *transpersonal aspects*, that is, feelings of wholeness or connectedness of all things. Factor 3 explained 3% of the variance and included items on *inspiration and creativity*. Factor 4 explained 3% of the variance and included items on *interpersonal relationships* and increased connectedness to other people. Factor 5 explained 2% of the variance and comprised items about an enhanced *relationship to nature*.

Factor 1 includes eight items, whereas the other factors include four items. The decision to expand the number of items in factor 1, aimed at a comprehensive representation of its thematic depth, was driven by the recognized significance of vitality within the afterglow phenomenon. This recognition was supported by previous anecdotal descriptions and the observation that vitality accounted for a substantial portion of the shared variance among the variables.

**Figure 1.**
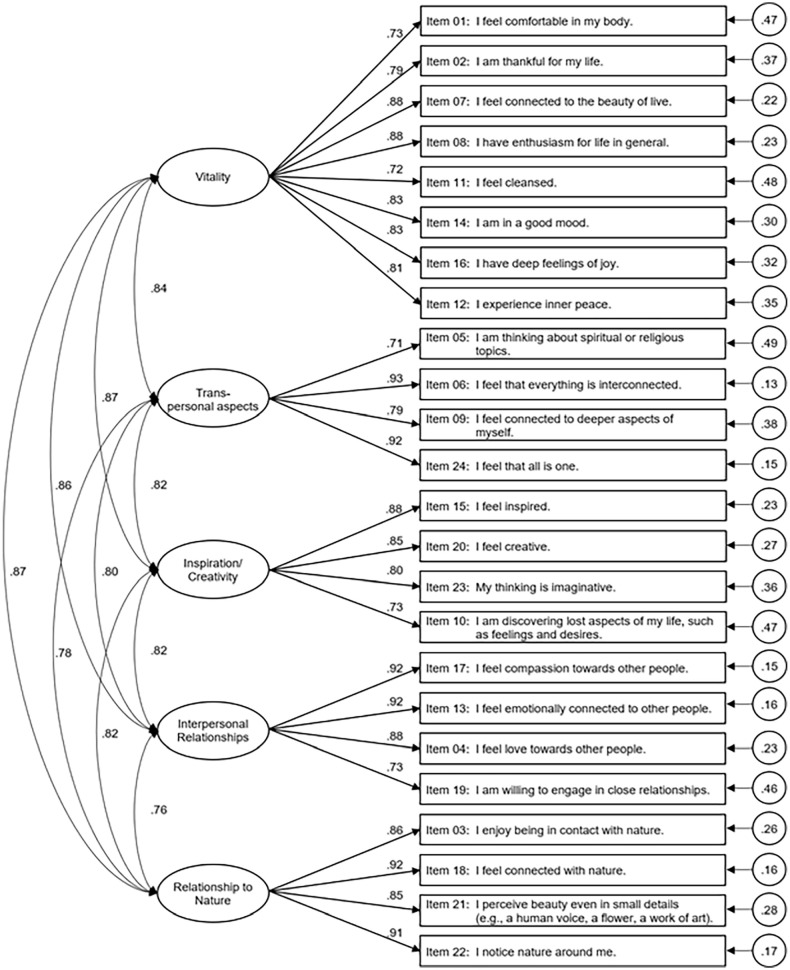
Summary of the CFA in the German psychedelic sample (*n* = 342). Ellipses represent the latent variables, and rectangles represent the manifest variables. Numbers next to long straight arrows are factor loadings. Circled numbers next to short straight arrows are residual variances. Numbers next to curved double-headed arrows are correlations among latent variables. All coefficients are standardized. CFA: confirmatory factor analysis.

The selected five-factor solution was replicated in the German psychedelic sample (*n* = 342) using CFA. Model fit was acceptable (RMSEA = 0.062 (90% CI = 0.055–0.069); CFI = 0.944; SRMR = 0.040). Figure 1 provides a summary of the CFA in the German sample. Descriptive statistics for the final selection of 24 items and internal consistencies of all factors are presented in Table 4. All scales had excellent internal consistency.

**Table 4.** Descriptive statistics and internal consistencies for the final selection of 24 AGI items in the psychedelic sample.

Factors (bold) and items	Mean (SD)				*t*	*p*	Cohen’s *d*	Cronbach’s alpha	
	English sample (*n* = 981)		German sample (*n* = 342)					English Sample	German Sample
AGI	63.24	(23.69)	56.63	(26.37)	4.10	<0.001	0.27	0.96	0.97
F1: Vitality	65.11	(24.59)	58.89	(27.29)	3.72	<0.001	0.23	0.92	0.94
I am in a good mood.	66.28	(29.94)	59.64	(31.11)					
I have enthusiasm for life in general	63.95	(30.36)	59.18	(32.93)					
I experience inner peace	62.59	(32.72)	55.83	(33.05)					
I am thankful for my life	73.77	(29.33)	69.64	(32.90)					
I feel comfortable in my body	63.28	(29.65)	56.97	(31.50)					
I feel connected to the beauty of life	68.62	(29.68)	62.12	(31.79)					
I feel cleansed	62.88	(32.82)	54.28	(34.40)					
I have deep feelings of joy	59.46	(33.11)	53.46	(33.28)					
F2: Transpersonal aspects	60.36	(28.81)	53.67	(30.54)	3.64	<0.001	0.22	0.87	0.90
I feel that everything is interconnected	64.90	(33.78)	54.36	(34.90)					
I feel that all is one	54.44	(36.22)	49.95	(34.86)					
I am thinking about spiritual or religious topics	56.66	(36.07)	55.72	(36.70)					
I feel connected to deeper aspects of myself	65.45	(30.15)	54.67	(32.87)					
F3: Inspiration/creativity	60.53	(26.90)	52.93	(28.75)	4.28	<0.001	0.27	0.85	0.89
I feel creative	61.21	(31.89)	53.13	(33.97)					
My thinking is imaginative	59.10	(32.62)	51.23	(33.98)					
I feel inspired	62.65	(31.00)	58.47	(31.63)					
I am discovering lost aspects of my life, such as feelings and desires	59.14	(33.66)	48.91	(33.39)					
F4: Interpersonal relationships	60.72	(27.95)	52.16	(29.59)	4.80	<0.001	0.29	0.91	0.92
I feel emotionally connected to other people	58.70	(31.11)	52.51	(31.63)					
I feel compassion toward other people	62.60	(30.19)	53.24	(31.76)					
I feel love toward other people	62.13	(31.70)	54.96	(32.95)					
I am willing to engage in close relationships	59.44	(33.37)	47.92	(35.78)					
F5: Relationship to nature	67.65	(27.95)	63.22	(30.48)	2.36	0.019	0.15	0.90	0.93
I notice nature around me	66.18	(32.16)	61.20	(32.79)					
I enjoy being in contact with nature	71.76	(31.06)	68.95	(33.08)					
I feel connected to nature	63.08	(32.67)	59.71	(33.43)					
I perceive beauty even in small details (e.g., a human voice, a flower, a work of art)	69.56	(31.18)	63.03	(34.19)					

AGI: Afterglow Inventory; SD: standard deviation; AGI: Final selection of 24 items.

### Validity assessment

A total number of 157 participants completed the AGS referring to an experience with one of the following non-psychedelic drugs: amphetamine (*n* = 61; 39%), methamphetamine (*n* = 12; 8%), cocaine (*n* = 57; 36%), or opioids (*n* = 27; 17%). Table 5 shows the sample characteristics of this control group. Differences between the psychedelic and non-psychedelic samples were observed for the variables of age, gender, education, and work situation. All differences had small effect sizes.

**Table 5.** Comparison of sample characteristics between the psychedelic and the non-psychedelic sample.

Sample characteristics	Psychedelic sample		Non-psychedelic control sample		*df*	*t* or χ²	*p*	Effect size^a^
	*N*	%	*N*	%				
	1323	100%	157	100%				
Age (SD)	29.84	(10.47)	27.83	(9.44)	198	2.45	0.015	0.194
Sex					2	7.50	0.024	0.073
Female	305	23.1	51	32.5				
Male	942	71.2	97	61.8				
Other	28	2.1	2	1.3				
Education					2	12.79	0.002	0.094
No degree	131	9.9	14	8.9				
Secondary school	550	41.6	88	56.1				
University	600	45.4	50	31.8				
Work situation					5	18.64	0.002	0.114
Student/apprentice	406	30.7	58	36.9				
Employed	517	39.1	45	28.7				
Self-employed/freelancer	156	11.8	10	6.4				
Unemployed	108	8.2	24	15.3				
Retired	28	2.1	4	2.5				
Other	66	5.0	11	7.0				

a Cohen’s *d* or Cramer’s *V*; SD: standard deviation.

**Figure 2.**
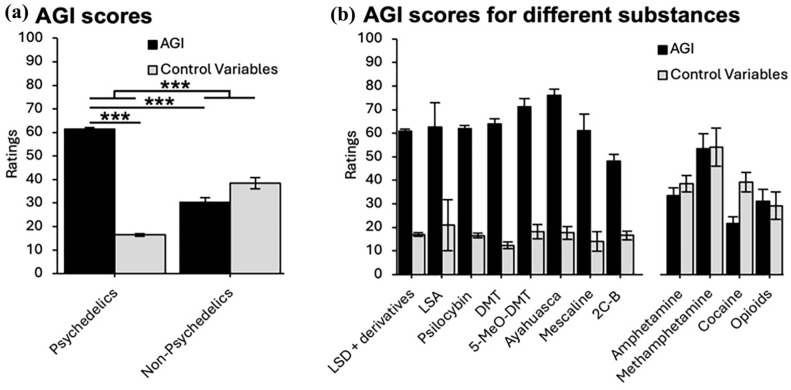
(a) Group-level AGI mean scores and scores on control variable for the psychedelic and non-psychedelic group (total sample, including English- and German-speaking participants). Significant differences between the main effects across the two groups in AGI and Control variables, as well as a significant interaction, promote validity. Error bars represent SEM. (b) Descriptive AGI mean scores and control variable scores plotted for different substances to display the consistency of the observed effects, and to show the variability in the data. Due to a lack of precise dosage information and variable participant numbers across the groups (see Table 6), statistical testing was omitted and data should not be considered to support quantitative differences between the strength of the afterglow effect across substances. Instead, the data are presented descriptively to support the consistency in differences between AGI scores and control variables across different psychedelics. These data shall inspire future research with larger samples and more precise dosage information to use the AGI for quantitative comparisons. AGI: Afterglow Inventory.

The mean scores of the final selection of the 24 items of the AGI and the five control items separately for each group are shown in Figure 2(a). Repeated measures ANOVA revealed a significant interaction of group and scale (*p* < 0.001; all statistics are presented in Supplemental Table S2). As expected, post hoc tests revealed that mean scores of the AGI were significantly higher in the psychedelics compared to the non-psychedelics group (mean difference = 31; *p* < 0.001). Furthermore, only in the psychedelics group, the mean scores of control items were significantly lower than the mean scores of the AGI (*p* < 0.001). A descriptive comparison of single items and factors between the psychedelic and non-psychedelic groups is displayed in Supplemental Table S3. The pronounced difference in mean values between the groups was also observed on the single-item level for all items. Figure 2(b) plots the mean scores of the AGI and control variables separately for each substance to descriptively display the consistency.

Lastly, the relationship of the AGI mean score with aspects of the acute drug experience was explored in the psychedelic group. A significant positive correlation between the mean score of the final AGI version and the retrospectively assessed subjective strength of the acute effect of the substance was observed (*r* = 0.165; *p* < 0.001). Furthermore, a significant positive correlation between the AGI mean score and the retrospective evaluation of the valence of the acute effects was observed (*r* = 0.251, *p* < 0.001). The more positive experiences were evaluated in retrospect, the higher the AGI mean score was. AGI mean scores separately for each psychedelic substance are shown in Table 6 and displayed in Figure 2(b). The most pronounced afterglow effects were measured in the ayahuasca group, whereas the weakest afterglow effects were observed in the 2C-B group.

**Table 6.** Mean AGI score for different psychedelic substances.

	Total sample			English sample			German sample		
	(*n* = 1323)			(*n* = 981)			(*n* = 342)		
	*n*	Mean	SD	*n*	Mean	SD	*n*	Mean	SD
LSD + derivates	607	60.88	(24.30)	429	62.37	(23.92)	178	57.29	(24.88)
LSA	9	62.63	(30.96)	3	70.22	(21.48)	6	58.83	(36.01)
Psilocybin	418	62.03	(24.27)	339	63.74	(23.17)	79	54.71	(27.51)
DMT	107	63.91	(24.34)	68	64.52	(23.17)	39	62.85	(23.82)
5-MeO-DMT	31	71.28	(19.25)	27	72.89	(14.87)	4	60.46	(40.12)
Ayahuasca	53	76.05	(18.30)	46	76.90	(17.64)	7	70.47	(22.99)
Mescaline	13	61.20	(25.35)	11	57.68	(25.96)	2	80.58	(9.07)
2C-B	85	48.11	(26.15)	58	50.73	(24.35)	27	42.48	(29.36)

5-MeO-DMT: 5-methoxy-DMT; DMT: N,N-dimethyltryptamine; LSA: lysergic acid amide; LSD: lysergic acid diethylamide; SD: standard deviation.

## Discussion

The present study aimed to develop a tool for the quantitative assessment of the “psychedelic afterglow” phenomenon. From our initial selection of 97 items, 24 were selected, loading onto 5 factors. These factors embrace (1) *Vitality*, including enhanced mood and well-being, (2) *Transpersonal aspects*, including feelings of wholeness and connectedness with oneself and other beings, (3) *Inspiration/creativity*, including experiences of enhanced imagination and inspiration, (4) *Interpersonal relationships*, including experiences of increased feelings of relatedness with others, and (5) *Relationship to nature*, including improved awareness of nature. These factors corroborate anecdotal descriptions of subacute afterglow phenomena (Pahnke, 1969) and findings on subacute effects of psychedelics reported in a systematic review (Evens et al., 2023). While some effects overlap with mood enhancement, the identified phenomena extend beyond a solely antidepressant effect, encompassing measurable aspects in both healthy individuals and those experiencing depression or other mental health problems.

The present study includes theory-driven item generation involving experienced experts, the development of parallel versions in English and German, and data-based reduction of items based on a large sample size in a subacute window after using psychedelics—thereby reducing memory bias often present in retrospective study designs.

Our sample was broadly international without relevant detected biases (education, previous consumption, and lifetime use), with a suitable sample size to validate a German and an English version, promoting the generalizability of the presented AGI structure.

### Index experience

The study design followed anecdotal reports on afterglow effects persisting up to 4 weeks (Pahnke et al., 1970), including participants only if they had used psychedelics no more than 28 days ago. It is a strength of our study that thereby we predominantly included participants with still prevailing subacute effects, avoiding retrospectively distorted memories of the effects. Nevertheless, only about 30% of participants reported still experiencing after-effects at the time they filled out the survey, whereas about 50% stated that after-effects had subsided within 14 days. This corroborates Pahnke’s observations that the total duration of afterglow phenomena exhibits temporal variability of “2 weeks to a month” (Pahnke et al., 1970). Similarly, another study observing afterglow effects in Native Americans regularly using peyote estimated the duration of afterglow effects to be between 7 and 10 days (Albaugh and Anderson, 1974). This feature of the data should not affect the revealed factor structure but inspires future research to explore differences in the duration of afterglow phenomena across different substances.

That 75% of participants reported having used LSD or psilocybin for the index experience may reflect epidemiological findings on the use of psychedelics (Evens et al., 2021; Winstock et al., 2021) that suggest that LSD and psilocybin are still the most commonly used psychedelics worldwide. By contrast, the other psychedelics exhibited relatively small sample sizes in our sample (see Table 6).

The observed amount of less than 2% of unpleasant index experiences can be considered relatively low compared to another study where 8.9% of participants reported functional impairment lasting longer than 1 day and 2.6% sought professional help (Simonsson et al., 2023). However, until today, reliable, setting-independent epidemiological data on the absolute rate of occurrence of unpleasant psychedelic experiences are still lacking. In our sample, 30% reported experiences that embraced both pleasant and unpleasant elements. These reports reflect the variability in psychedelic experiences and suggest the suitability of the sample for the performed analyses. This inspires future research to test for potential differences in afterglow experiences mediated by specific features of the index experience, such as pleasantness, mystical qualities, or other characteristics.

Regarding the non-psychedelic control group, more than half of the reported index experiences were with amphetamine-type stimulants (i.e., amphetamine or methamphetamine). Taken together, the sample appears to display no relevant biases and is suited for the factor analyses.

### Item and factor structure of the AGI

The first factor explained 54% of the variances, whereas the other four factors explained between 2% and 4% of the variance each. Factor 1 (Vitality) encompasses emotional and motivational aspects, such as effects on mood and drive, which show some overlap with antidepressant effects in people with depressive syndromes, without being limited to them. In addition to mood-related effects, however, there are also positive attitudes toward life, gratitude, and feelings of peace and cleansing. By contrast, Factors 2–5 show significantly less overlap with antidepressant effects. Factor 2 (Transpersonal aspects) includes experiences that resemble so-called mystical experiences, as have been repeatedly described under the acute effects of psychedelics (Griffiths et al., 2006; Pahnke, 1969). An enhanced sense of connectedness is also reflected in this factor, even after the acute experience has subsided. Factor 3 (Inspiration/creativity) encompasses the subjective experience of increased creativity, an effect that has been previously reported after the use of psychedelics (Mason et al., 2021), particularly during the subacute window (Basedow et al., 2021; Evens et al., 2023).

Factor 4 (Interpersonal relationships) includes experiences of heightened feelings of connection with other people, which have also been repeatedly reported (Kettner et al., 2021). Factor 5 (Relationship to nature) describes feelings of connectedness with nature, including a joyful engagement with and perception of natural phenomena. Effects of psychedelic use on nature-relatedness have been reported in several studies (Aday et al., 2024; Kettner et al., 2019).

Thus, Factors 2, 4, and 5 encompass various aspects of connectedness—whether in relation to other people, nature, or spiritual or religious experiences, as similarly reflected in questionnaires that attempt to capture acute experiences of connectedness under psychedelics (Watts et al., 2022). These factors may therefore also be considered as different dimensions of a generally heightened sense of connectedness with life itself, as described in mystical experiences independent of psychedelic use. For instance, the philosopher and theologist Martin Buber similarly describes moments of encounter with other people, nature, or metaphysical entities as different dimensions of being connected (Buber, 1972).

The factors of the AGI can thus be divided into experiences that focus more on intrapsychic experiences (Factors 1 and 3) and those that represent feelings of connectedness. In this way, they appear to echo, in the subacute phase of substance effects, phenomenological areas that are typically—albeit often more intensely—described in the acute phase of substance effects.

### Validity

As expected, the psychedelic group exhibited significantly higher AGI scores than the non-psychedelic control group (see Figure 2), suggesting that AGI items reflect the subacute effects of psychedelics more specifically than the effects of the control substances. We also administered control items including negative subacute effects which were not expected to characterize psychedelic subacute effects, but rather negative subacute effects. As anticipated, only in the psychedelics group were the mean scores of control items significantly lower than the mean scores of the AGI, corroborating the validity of the discriminative features of the AGI regarding psychedelic and non-psychedelic drugs. Despite the dominance of index experiences with LSD and psilocybin, the consistency in the differences between AGI score and control items across substances (see Figure 2) supports the validity of the AGI across different psychedelic substances. However, since our sample included relatively little data on the effects of psychedelics other than LSD and psilocybin, further studies are needed to promote the generalizability and validity of the findings for other psychedelics.

Anecdotal reports suggest differences in the ability and potency of specific psychedelics to facilitate afterglow effects. For instance, substances like 5-MeO-DMT have anecdotally been associated with pronounced subacute effects. However, it has not yet been systematically investigated if and how the strength of afterglow effects varies across substances, and trip reports (e.g., on Erowid, https://www.erowid.org) do not suffice as data references due to diverse biases.

In the current sample, we found a significant correlation between the intensity and the degree of positive appraisal of the index experience and the strength of the afterglow effects, further supporting the validity of our afterglow quantification. This finding might be particularly relevant as it is still debated whether the quality and intensity of acute psychedelic effects predict individual post-acute outcomes (Majić et al., 2015; Roseman et al., 2018; Smigielski et al., 2019). For instance, several studies have suggested that mystical experiences during the acute effects of the substance predict subacute and long-term effects (Griffiths et al., 2008). Notably, high measures of afterglow effects are not per se identical with positive therapy prognoses or outcomes. Furthermore, the appraisal of the valence of acute effects might retrospectively be affected by the valence of the outcome, not allowing causal attributions.

### Limitations

Recruiting participants for anonymous online studies is associated with an increased risk of selecting a biased sample, potentially resulting in non-representative samples that cannot be generalized to the population of interest, in this case, psychedelic users. Notably, our sample predominantly consisted of relatively young, highly educated men experienced with psychedelics and other substances. Hence, findings may only partly generalize to populations with different sample characteristics. As our study aimed at the construction and initial validation of a new questionnaire, it does not serve to make quantitative claims, with regards to strength or duration of afterglow effects across substances or different sample characteristics. These may be explored in future studies.

Finally, it is important to acknowledge that the psychedelic afterglow might be a typical psychedelic-associated phenomenon, but not the only set of experiences that may be observed after the use of psychedelics. Despite acknowledging the possibility of negative subacute effects, we opted to align with classical descriptions of the “afterglow,” given that those phenomena exhibit a high specificity for psychedelics. Future studies are needed to systematically study negative subacute effects.

The majority of studies validating new instruments for assessing phenomena related to non-ordinary states of consciousness have been conducted in one language and then translated into others. In this study, we validated both the English and German versions of the questionnaire simultaneously, finding a high degree of consistency between the two versions. Nonetheless, the German participants scored significantly lower on the mean AGI total scores and the mean scores for each AGI factor. Interestingly, similar findings have been reported in previous studies (Wolff et al., 2022, 2024), indicating potential cultural differences between German- and English-speaking samples regarding response behavior.

### Implications for future research

Quantifying afterglow phenomena with the AGI may be valuable for several reasons. To our knowledge, the AGI is the first available instrument specifically designed to assess the subacute effects of psychedelics. It could serve as a tool for comparing different classic psychedelics in terms of their ability to facilitate afterglow effects in future studies. A quantitative estimation of these afterglow effects and their comparison across studies could be useful, as it might serve as a tool for estimating the potential of a substance, dosage, or intervention to induce beneficial subacute effects. Future research should investigate factors influencing the intensity of the subacute afterglow. Several factors need to be considered when quantifying after-effects in the subacute window, which may not have been balanced between sub-samples, such as (1) the length of time between index experience and assessment, as the natural course of post-acute effects may vary over time; (2) dosage, intensity, and valence of the preceding acute psychedelic experience; and (3) the mindset of the user and the setting in which the index experience occurred.

In addition, the AGI could be used to assess the intensity and duration of the post-psychedelic window of change, where psychotherapeutic interventions have been suggested to be particularly effective (Evens et al., 2023). The “afterglow” phenomenon can be observed during a timeframe when biological mechanisms and psychological dimensions of the experiences gradually disentangle and wear off. Therapy sessions following psychedelic experiences have been to maximize benefits, minimize risks and “integrate” the experiences into everyday life, techniques which have been labeled as “psychedelic integration” by different authors (Bathje et al., 2022; Gorman et al., 2021; Grof, 1979), for a comprehensive overview, see the book by Marc Aixalà on Psychedelic Integration (Aixalà, 2022). The AGI could contribute to exploring mechanisms of post-psychedelic change and evaluating the relationship between subacute effects and therapeutic techniques applied during this period. These questions are crucial for the use of psychedelics in therapy, as integration therapy aims to transform subacute psychological insights from acute psychedelic experiences into lasting change.

The AGI could also be applied to investigate subacute states following non-substance-related experiences. Notably, acute experiences made under psychedelics tend to be profoundly meaningful for users (Griffiths et al., 2006), and long-term positive outcomes might be associated with these experiential features (Griffiths et al., 2008). Post-psychedelic effects may overlap phenomenologically with outcomes from existential or spiritual experiences unrelated to psychotropic substances. Examples include the birth of a child, falling in love, or intense spiritual practices which are associated with emotional development and personal growth. Other non-drug-related experiences that induce non-ordinary states of consciousness, such as immersive breathing (Uthaug et al., 2022) or flicker light (Montgomery et al., 2024), may show experiential similarities with psychedelic experiences. The AGI could help identify differences and overlaps between “afterglows” following existential experiences with and without the use of psychedelics or other substances.

When asking participants retrospectively about acute psychedelic drug effects, positive memories might be reactivated, potentially altering demand characteristics. This could confound AGI effects, making it difficult to disentangle pharmacological from experiential effects. In addition, it remains to be established how both pharmacodynamic and psychological dimensions contribute distinctively to the afterglow phenomenon.

Recent studies suggest that subacute psychedelic phenomena may reflect biological mechanisms, such as increased neuroplasticity (Olson, 2022) or enhanced brain connectivity (Gattuso et al., 2023). The AGI could be useful in assessing the phenomenological correlates of these biological mechanisms and investigating the role of subjective subacute experiences in therapy with psychedelics. In addition, future research questions might explore the relevance of set and setting in relation to the intensity and duration of afterglow effects. For example, the AGI could be used to evaluate potential influences of individual characteristics on the likelihood of experiencing afterglow effects. These characteristics may include personality structure, prior experiences with psychedelics, expectancy factors, and the presence of psychopathological symptoms. Finally, the AGI could also serve as a tool for modeling predictor variables for long-term outcomes.

In this context, the concept of a “psychedelic afterglow” primarily encompasses positive effects during the “carryover” period. This contrasts with most other psychoactives, where predominantly negative (or “hangover”) effects are reported during the subacute period. However, it is important to note that some individuals report unpleasant or challenging after-effects (Simonsson et al., 2023), which might also be specific to psychedelics. This aspect warrants future investigation, as research on negative or adverse post-acute effects of psychedelics remains limited, with only a few recent studies addressing this issue (Bremler et al., 2023; Evans et al., 2023).

## Conclusions

Here we present the AGI, a new tool for assessing typical subacute (“afterglow” or “carryover”) effects associated with psychedelics. The AGI has been simultaneously developed and is available in both English and German. Based on data from an international, anonymous online survey a five-factor solution with 24 items provided the best fit for the data. The five factors are as follows: (1) Vitality, (2) Transpersonal aspects, (3) Inspiration/creativity, (4) Interpersonal relationships, and (5) Relationship to nature. All scales demonstrated excellent internal consistency. Importantly, the AGI items effectively differentiated between the psychedelic group and a non-psychedelic control group using stimulants and opioids. In addition, the AGI total score correlated with the intensity and valence of the preceding acute psychedelic experience, with more intense and positive experiences predicting higher AGI scores. The AGI may serve as a tool for quantitatively assessing and comparing specific psychological effects observed during the subacute phase of psychedelic drug actions. Since afterglow phenomena have been linked to increased openness for interpersonal encounters and psychotherapeutic interventions, the AGI could be valuable in future studies exploring the connection between acute psychedelic experiences and post-acute outcomes.

## Supplemental Material

sj-docx-1-jop-10.1177_02698811251326937 – Supplemental material for The Afterglow Inventory (AGI): Validation of a new instrument for measuring subacute effects of classic serotonergic psychedelics Supplemental material, sj-docx-1-jop-10.1177_02698811251326937 for The Afterglow Inventory (AGI): Validation of a new instrument for measuring subacute effects of classic serotonergic psychedelics by Tomislav Majić, Timo Torsten Schmidt, Anna Gröticke, Peter Gasser, William A Richards, Thomas G Riemer and Ricarda Evens in Journal of Psychopharmacology
