# Supplementary material for: The Afterglow Inventory (AGI): Validation of a new instrument for measuring subacute effects of classic serotonergic psychedelics
Source: J Psychopharmacol. 2025 Mar 31;39(5):474–88. doi: 10.1177/02698811251326937 (PMC12099019; doi:10.1177/02698811251326937)
Supplement: sj-docx-1-jop-10.1177_02698811251326937 – Supplemental material for The Afterglow Inventory (AGI): Validation of a new instrument for measuring subacute effects of classic serotonergic psychedelics [file sj-docx-1-jop-10.1177_02698811251326937.docx]

**Supplementary material**

[**Table s1. List of initial 97 items of the AGI**](#_Toc154744758)

[**Table s2. Repeated measures ANOVA Scale*Group**](#_Toc154744759)

[**Table s3. Comparison of final selection of 24 AGI items between psychedelic and non-psychedelic group**](#_Toc154744760)

[**Appendix A: Printable version of the AGI (English version)**](#_Toc154744761)

[**Appendix B: Printable version of the AGI (German version)**](#_Toc154744762)

| **Table s1. List of initial 97 items of the AGI (items of the final AGI version in bold).** | | |
| --- | --- | --- |
|  | **English** | **German** |
| 1. | I live consciously in the present moment. | Ich lebe bewusst im gegenwärtigen Moment. |
| **2.** | **I feel comfortable in my body.** | **Ich fühle mich in meinem Körper wohl.** |
| 3. | I feel connected to my intuition. | Ich fühle mich mit meiner Intuition verbunden. |
| 4. | I am sensitive to the suffering of other people. | Ich bin sensibel für das Leiden anderer Menschen. |
| 5. | I feel a need to improve existing relationships. | Ich habe das Bedürfnis, bestehende Beziehungen zu verbessern. |
| 6. | I am honest with myself. | Ich bin ehrlich zu mir selbst. |
| 7. | I can see my weaknesses clearly. | Ich kann meine Schwächen klar erkennen. |
| **8.** | **I am thankful for my life.** | **Ich bin dankbar für mein Leben.** |
| 9. | I am relaxed. | Ich bin entspannt. |
| 10. | It is easy for me to refrain from things that are harmful to me. | Es fällt mir leicht, Dinge zu unterlassen, die schädlich für mich sind. |
| 11. | I feel connected to my body. | Ich fühle mich mit meinem Körper verbunden. |
| 12. | It is easy for me to become immersed in things (e.g., movies, theatre, nature, voices, past events). | Es fällt mir leicht, mich in Dinge zu vertiefen (z.B. Filme, Theater, Natur, Stimmen, frühere Ereignisse). |
| **13.** | **I enjoy being in contact with nature.** | **Ich genieße es, im Kontakt mit der Natur zu sein.** |
| 14. | I am benevolent towards other people. | Ich bin wohlwollend gegenüber anderen Menschen. |
| 15. | I have a positive relationship to other people. | Ich habe eine positive Beziehung zu anderen Menschen. |
| 16. | I have clarity of mind. | Ich habe Klarheit im Denken. |
| 17. | I am benevolent with myself. | Ich bin mir selbst gegenüber wohlwollend. |
| 18. | I feel humble. | Ich spüre Demut. |
| 19. | I am full of energy. | Ich bin voller Energie. |
| 20. | I am motivated to tackle personal goals. | Ich bin motiviert persönliche Ziele anzugehen. |
| 21. | I perceive things the way they really are. | Ich nehme die Dinge so wahr, wie sie wirklich sind. |
| 22. | I experience music with intensity. | Ich erlebe Musik intensiv. |
| 23. | I feel one with everything around me. | Ich fühle mich eins mit allem um mich herum. |
| 24. | I am patient with other people. | Ich bin geduldig mit anderen Menschen. |
| 25. | I enjoy social interactions. | Ich habe Freude an sozialen Interaktionen. |
| **26.** | **I am thinking about spiritual or religious topics.** | **Ich denke über spirituelle oder religiöse Themen nach.** |
| 27. | I am patient with myself. | Ich bin geduldig mit mir selbst. |
| 28. | I have appreciation for life. | Ich habe Wertschätzung für das Leben. |
| 29. | My life feels playful. | Mein Leben fühlt sich spielerisch an. |
| 30. | I take responsibility for my life. | Ich übernehme Verantwortung für mein Leben. |
| 31. | My mind and my emotions are connected. | Mein Verstand und meine Gefühle sind verbunden. |
| 32. | I can recollect some past experiences in my life with great clarity. | Ich kann mich mit großer Klarheit an einige frühere Erfahrungen in meinem Leben erinnern. |
| **33.** | **I feel that everything is interconnected.** | **Ich fühle, dass alles miteinander verbunden ist.** |
| 34. | I have a good sense of what is going on between other people. | Ich habe ein gutes Gespür dafür, was zwischen anderen Menschen vor sich geht. |
| 35. | I feel close to my family and friends. | Ich empfinde Nähe zu meiner Familie und Freunden. |
| 36. | I am able to deal with unpleasant issues. | Ich bin in der Lage, mich mit unangenehmen Themen auseinanderzusetzen. |
| 37. | I am optimistic. | Ich bin optimistisch. |
| **38.** | **I feel connected to the beauty of life.** | **Ich fühle mich mit der Schönheit des Lebens verbunden.** |
| **39.** | **I have enthusiasm for life in general.** | **Ich habe Begeisterung für das Leben im Allgemeinen.** |
| 40. | I need little sleep. | Ich brauche wenig Schlaf. |
| **41.** | **I feel connected to deeper aspects of myself.** | **Ich fühle mich mit tieferen Aspekten von mir selbst verbunden.** |
| 42. | I experience sexuality as enjoyable. | Ich erlebe Sexualität als genussvoll. |
| **43.** | **I feel that all is one.** | **Ich fühle, dass alles eins ist.** |
| 44. | I have compassion for my family and friends. | Ich empfinde Mitgefühl für meine Familie und Freunde. |
| 45. | I feel mentally flexible. | Ich fühle mich mental flexibel. |
| 46. | I am free from rumination. | Ich bin frei von sorgenvollem Grübeln. |
| 47. | I face death without fear. | Ich schaue dem Tod ohne Angst entgegen. |
| 48. | I feel attuned to what really matters. | Ich bin eingestellt auf das, was wirklich zählt. |
| **49.** | **I feel cleansed.** | **Ich fühle mich gereinigt.** |
| 50. | I feel desire for sex. | Ich verspüre den Wunsch nach Sex. |
| 51. | I feel connected to my true self | Ich fühle mich mit meinem wahren Selbst verbunden. |
| 52. | I can look at stressful life situations without having to react immediately. | Ich kann belastende Lebenssituationen betrachten, ohne sofort reagieren zu müssen. |
| **53.** | **I feel compassion towards other people.** | **Ich habe Mitgefühl mit anderen Menschen.** |
| **54.** | **I feel emotionally connected to other people.** | **Ich fühle mich emotional mit anderen Menschen verbunden.** |
| 55. | I am open to new ideas. | Ich bin offen für neue Ideen. |
| 56. | I am able to interrupt pointless rumination. | Ich bin in der Lage sinnloses Grübeln zu unterbrechen. |
| 57. | I am confident that my life will be good. | Ich bin zuversichtlich, dass mein Leben gut wird. |
| **58.** | **I am in a good mood.** | **Ich bin in einer guten Stimmung.** |
| 59. | I am free from feelings of guilt. | Ich bin frei von Schuldgefühlen. |
| 60. | I perceive my sensory impressions with intensity. | Ich nehme meine Sinneseindrücke intensiv wahr. |
| 61. | I face challenges calmly. | Ich sehe Herausforderungen gelassen entgegen. |
| 62. | I am sensitive to other people's needs. | Ich bin sensibel für die Bedürfnisse anderer Menschen. |
| **63.** | **I feel love towards other people.** | **Ich empfinde Liebe zu anderen Menschen.** |
| 64. | It is easy for me to look at things from different perspectives. | Mir fällt es leicht, die Dinge aus unterschiedlichen Perspektiven zu betrachten. |
| 65. | I am open to going beyond the world of reason and to experience something entirely new. | Ich bin offen dafür, die Welt der Vernunft zu überschreiten und etwas ganz Neues zu erleben. |
| 66. | I feel confident interacting with other people. | Ich fühle mich sicher im Umgang mit anderen Menschen. |
| 67. | I enjoy my life. | Ich genieße mein Leben. |
| 68. | My mood changes a lot. | Meine Stimmung wechselt häufig. |
| 69. | I feel a need for healthy food. | Ich habe ein Bedürfnis nach gesundem Essen. |
| 70. | I can fully experience emotions, whether positive or negative. | Ich kann Emotionen vollständig erleben, ob positiv oder negativ. |
| 71. | I understand other people's problems. | Ich verstehe die Probleme anderer Menschen. |
| 72. | I feel connected to humanity. | Ich fühle mich mit der Menschheit verbunden. |
| **73.** | **I feel inspired.** | **Ich fühle mich inspiriert.** |
| 74. | I can see my mistakes clearly. | Ich kann meine Fehler klar erkennen. |
| 75. | I have clarity about what is really important for me in life. | Ich habe Klarheit darüber, was für mir wirklich wichtig ist im Leben. |
| 76. | I feel love. | Ich spüre Liebe. |
| **77.** | **I have deep feelings of joy.** | **Ich empfinde tiefe Gefühle von Freude.** |
| 78. | I clearly perceive the taste of food. | Ich nehme den Geschmack von Essen deutlich wahr. |
| **79.** | **I feel connected to nature.** | **Ich fühle mich mit der Natur verbunden.** |
| 80. | I am tolerant of other people. | Ich bin anderen Menschen gegenüber tolerant. |
| **81.** | **I am willing to engage in close relationships.** | **Ich bin bereit, mich auf enge Beziehungen einzulassen.** |
| **82.** | **I feel creative.** | **Ich fühle mich kreativ.** |
| 83. | I accept myself as I am. | Ich akzeptiere mich so wie ich bin. |
| 84. | I sense meaning in life. | Ich spüre Sinn im Leben. |
| **85.** | **I experience inner peace.** | **Ich erlebe inneren Frieden.** |
| **86.** | **I perceive beauty even in small details (e.g., a human voice, a flower, a work of art).** | **Ich nehme Schönheit auch in kleinen Details wahr (z.B. einer menschlichen Stimme, einer Blume, einem Kunstwerk).** |
| 87. | I enjoy tasty food. | Ich genieße leckeres Essen. |
| **88.** | **I notice nature around me.** | **Ich nehme die Natur um mich herum wahr.** |
| 89. | I care about other people. | Ich sorge mich um andere Menschen. |
| 90. | I am willing to engage in new relationships. | Ich bin bereit, mich auf neue Beziehungen einzulassen. |
| **91.** | **My thinking is imaginative.** | **Mein Denken ist phantasievoll.** |
| 92. | I have compassion towards myself. | Ich habe mir selbst gegenüber Mitgefühl. |
| **93.** | **I am discovering lost aspects of my life, such as feelings and desires.** | **Ich entdecke verloren gegangene Aspekte meines Lebens, wie Gefühle und Wünsche.** |
| 94. | I feel ease in my life. | Ich spüre Leichtigkeit in meinem Leben. |
| 95. | I manage not to follow bad habits. | Ich schaffe es, schlechten Gewohnheiten nicht nachzugehen. |
| 96. | I enjoy physical contact with people I like. | Ich genieße den körperlichen Kontakt mit Menschen, die ich mag. |
| 97. | I am aware of life in all things. | Ich bin mir des Lebens in allen Dingen bewusst. |

| **Table s2. Repeated measures ANOVA Scale*Group** | | | |
| --- | --- | --- | --- |
|  | **df** | **F** | **p** |
| Scale | 1,1478 | 221 | <0.001 |
| Group | 1,1478 | 11 | 0.001 |
| Scale*Group | 1,1478 | 453 | <0.001 |
| Repeated measures analysis of variance (ANOVA) with the within-group factor “scale” (mean score of the final AGI selection vs. mean score of control items) and the between-group factor “group” (psychedelic vs. non-psychedelic groups) | | | |
|  |  |  |  |

| **Table s3. Comparison of final selection of 24 AGI items between psychedelic and non-psychedelic group** | | | | | | | |
| --- | --- | --- | --- | --- | --- | --- | --- |
| **Factors (bold) and items** | **Mean (SD)** | | | | **t** | **p** | **Cohen’s d** |
|  | **Psychedelic**  **(n = 1,323)** | | **Non-Psychedelic**  **(n = 157)** | |  |  |  |
|  |  | |  | |  |  |  |
| **AGI** | 61.53 | (24.57) | 30.33 | (24.97) |  | <0.001 | 1.26 |
|  |  |  |  |  |  |  |  |
| **F1: Vitality** | 63.50 | (25.45) | 30.29 | (26.50) | -15.40 | <0.001 | 1.28 |
| I am in a good mood. | 64.57 | (30.38) | 32.69 | (33.47) |  |  |  |
| I have enthusiasm for life in general | 62.72 | (31.10) | 32.85 | (33.55) |  |  |  |
| I experience inner peace | 60.85 | (32.92) | 25.51 | (30.11) |  |  |  |
| I am thankful for my life | 72.70 | (30.34) | 40.25 | (36.84) |  |  |  |
| I feel comfortable in my body | 61.65 | (30.25) | 32.68 | (33.81) |  |  |  |
| I feel connected to the beauty of life | 66.94 | (30.36) | 27.75 | (31.37) |  |  |  |
| I feel cleansed | 60.66 | (33.43) | 19.72 | (27.80) |  |  |  |
| I have deep feelings of joy | 57.91 | (33.25) | 30.83 | (34.63) |  |  |  |
|  |  |  |  |  |  |  |  |
| **F2: Transpersonal aspects** | 58.63 | (29.40) | 25.49 | (26.49) | -14.64 | <0.001 | 1.18 |
| I feel that everything is interconnected | 62.18 | (34.37) | 27.07 | (33.80) |  |  |  |
| I feel that all is one | 53.28 | (35.91) | 20.61 | (27.67) |  |  |  |
| I am thinking about spiritual or religious topics | 56.41 | (36.22) | 25.25 | (33.02) |  |  |  |
| I feel connected to deeper aspects of myself | 62.66 | (31.22) | 29.01 | (32.02) |  |  |  |
|  |  |  |  |  |  |  |  |
| **F3: Inspiration/Creativity** | 58.56 | (27.58) | 31.47 | (28.60) | -11.59 | <0.001 | 0.96 |
| I feel creative | 59.12 | (32.62) | 30.87 | (33.98) |  |  |  |
| My thinking is imaginative | 57.07 | (33.15) | 31.94 | (35.25) |  |  |  |
| I feel inspired | 61.57 | (31.20) | 29.57 | (31.48) |  |  |  |
| I am discovering lost aspects of my life, such as feelings and desires | 56.49 | (33.88) | 33.50 | (33.64) |  |  |  |
|  |  |  |  |  |  |  |  |
| **F4: Interpersonal Relationships** | 58.50 | (28.62) | 31.64 | (30.07) | -11.06 | <0.001 | 0.92 |
| I feel emotionally connected to other people | 57.10 | (31.35) | 30.24 | (31.59) |  |  |  |
| I feel compassion towards other people | 60.18 | (30.86) | 32.88 | (34.73) |  |  |  |
| I feel love towards other people | 60.27 | (32.16) | 31.97 | (32.86) |  |  |  |
| I am willing to engage in close relationships | 56.46 | (34.37) | 31.46 | (35.56) |  |  |  |
|  |  |  |  |  |  |  |  |
| **F5: Relationship to Nature** | 66.50 | (28.68) | 32.84 | (30.00) | -13.84 | <0.001 | 1.15 |
| I notice nature around me | 64.89 | (32.38) | 31.65 | (34.94) |  |  |  |
| I enjoy being in contact with nature | 71.03 | (31.60) | 37.99 | (36.50) |  |  |  |
| I feel connected to nature | 62.20 | (32.89) | 25.73 | (31.57) |  |  |  |
| I perceive beauty even in small details (e.g., a human voice, a flower, a work of art) | 67.87 | (32.10) | 36.00 | (36.91) |  |  |  |
| SD: standard deviation | | | | | | | |

# **Appendix A: Printable version of the AGI (English version)**

**Afterglow Inventory (AGI)**

In the following questionnaire you will find a number of statements about your thoughts and feelings. Below each statement there is a line with the two endpoints **"NO, not more than usual"** and **"YES, much more than usual"**.

Please judge to what extent the statements apply to **your experience during the days following your last consumption of the substance as compared to your normal/everyday experience**.

Accordingly, make a vertical mark on the corresponding point on the line below the statement.

Please note that the rating **"NO, not more than usual"** corresponds to a **mark at the very left of the scale**.

**Please use all intermediate gradations to most accurately describe your experiences!**

1. I feel comfortable in my body.

NO, not more than usual

YES, much more than usual

1. I am thankful for my life.

NO, not more than usual

YES, much more than usual

1. I enjoy being in contact with nature.

NO, not more than usual

YES, much more than usual

1. I feel love towards other people.

NO, not more than usual

YES, much more than usual

1. I am thinking about spiritual or religious topics.

NO, not more than usual

YES, much more than usual

1. I feel that everything is interconnected.

NO, not more than usual

YES, much more than usual

1. I feel connected to the beauty of life.

NO, not more than usual

YES, much more than usual

1. I have enthusiasm for life in general.

NO, not more than usual

YES, much more than usual

1. I feel connected to deeper aspects of myself.

NO, not more than usual

YES, much more than usual

1. I am discovering lost aspects of my life, such as feelings and desires.

NO, not more than usual

YES, much more than usual

1. I feel cleansed.

NO, not more than usual

YES, much more than usual

1. I experience inner peace.

NO, not more than usual

YES, much more than usual

1. I feel emotionally connected to other people.

NO, not more than usual

YES, much more than usual

1. I am in a good mood.

NO, not more than usual

YES, much more than usual

1. I feel inspired.

NO, not more than usual

YES, much more than usual

1. I have deep feelings of joy.

NO, not more than usual

YES, much more than usual

1. I feel compassion towards other people.

NO, not more than usual

YES, much more than usual

1. I feel connected to nature.

NO, not more than usual

YES, much more than usual

1. I am willing to engage in close relationships.

NO, not more than usual

YES, much more than usual

1. I feel creative.

NO, not more than usual

YES, much more than usual

1. I perceive beauty even in small details (e.g., a human voice, a flower, a work of art).

NO, not more than usual

YES, much more than usual

1. I notice nature around me.

NO, not more than usual

YES, much more than usual

1. My thinking is imaginative.

NO, not more than usual

YES, much more than usual

1. I feel that all is one.

NO, not more than usual

YES, much more than usual

**Afterglow Inventory (AGI) Scoring Instructions**

Visual analogue scales (VAS) printed on DIN A4 paper (210 x 297 mm) should be precisely 100 mm long. Item scores range from 0 to 100. Each item score is read out by measuring the horizontal distance between the left endpoint of the VAS and the position marked by the test subject in mm. Scores on the main scale and subscales are calculated as follows.

Subscales:

| **Vitality** | = (Item 01 + Item 02 + Item 07 + Item 08 + Item 11 + Item 12  + Item 14 + Item 16) / 8 |
| --- | --- |
| **Transpersonal Aspects** | = (Item 05 + Item 06 + Item 09 + Item 24) / 4 |
| **Inspiration/Creativity** | = (Item 10 + Item 15 + Item 20 + Item 23) / 4 |
| **Interpersonal Relationships** | = (Item 04 + Item 13 + Item 17 + Item 19) / 4 |
| **Relationship to Nature** | = (Item 03 + Item 18 + Item 21 + Item 22) / 4 |

Main Scale:

| **AGI mean score** | = Sum of all 24 items / 24 |
| --- | --- |

# **Appendix B: Printable version of the AGI (German version)**

**Afterglow Inventory (AGI)**

In diesem Fragebogen finden Sie eine Reihe von Aussagen zu Ihren Gedanken und Gefühlen. Unter jeder Aussage befindet sich eine Linie mit den zwei Endpunkten **"NEIN, nicht mehr als gewöhnlich”** und **“JA, sehr viel mehr als gewöhnlich"**.

Bitte beurteilen Sie, inwieweit die Aussagen auf Ihr Erleben **in der letzten Woche im Vergleich zu Ihrem gewöhnlichen/alltäglichen Erleben** zutreffen.

Markieren Sie dazu auf der jeweils darunterliegenden Linie die entsprechende Stelle mit einem **senkrechten Strich**.

Bitte beachten Sie, dass die Einschätzung **"NEIN, nicht mehr als gewöhnlich"** einem **Strich genau links auf dem Endpunkt** entspricht.

**Bitte benutzen Sie alle Zwischenstufen, um Ihr Erleben möglichst genau zu beschreiben!**

1. Ich fühle mich in meinem Körper wohl.

NEIN, nicht mehr als gewöhnlich

JA, sehr viel mehr als gewöhnlich

1. Ich bin dankbar für mein Leben.

NEIN, nicht mehr als gewöhnlich

JA, sehr viel mehr als gewöhnlich

1. Ich genieße es, im Kontakt mit der Natur zu sein.

NEIN, nicht mehr als gewöhnlich

JA, sehr viel mehr als gewöhnlich

1. Ich empfinde Liebe zu anderen Menschen.

NEIN, nicht mehr als gewöhnlich

JA, sehr viel mehr als gewöhnlich

1. Ich denke über spirituelle oder religiöse Themen nach.

NEIN, nicht mehr als gewöhnlich

JA, sehr viel mehr als gewöhnlich

1. Ich fühle, dass alles miteinander verbunden ist.

NEIN, nicht mehr als gewöhnlich

JA, sehr viel mehr als gewöhnlich

1. Ich fühle mich mit der Schönheit des Lebens verbunden.

NEIN, nicht mehr als gewöhnlich

JA, sehr viel mehr als gewöhnlich

1. Ich habe Begeisterung für das Leben im Allgemeinen.

NEIN, nicht mehr als gewöhnlich

JA, sehr viel mehr als gewöhnlich

1. Ich fühle mich mit tieferen Aspekten von mir selbst verbunden.

NEIN, nicht mehr als gewöhnlich

JA, sehr viel mehr als gewöhnlich

1. Ich entdecke verloren gegangene Aspekte meines Lebens, wie Gefühle und Wünsche.

NEIN, nicht mehr als gewöhnlich

JA, sehr viel mehr als gewöhnlich

1. Ich fühle mich gereinigt.

NEIN, nicht mehr als gewöhnlich

JA, sehr viel mehr als gewöhnlich

1. Ich erlebe inneren Frieden.

NEIN, nicht mehr als gewöhnlich

JA, sehr viel mehr als gewöhnlich

1. Ich fühle mich emotional mit anderen Menschen verbunden.

NEIN, nicht mehr als gewöhnlich

JA, sehr viel mehr als gewöhnlich

1. Ich bin in einer guten Stimmung.

NEIN, nicht mehr als gewöhnlich

JA, sehr viel mehr als gewöhnlich

1. Ich fühle mich inspiriert.

NEIN, nicht mehr als gewöhnlich

JA, sehr viel mehr als gewöhnlich

1. Ich empfinde tiefe Gefühle von Freude.

NEIN, nicht mehr als gewöhnlich

JA, sehr viel mehr als gewöhnlich

1. Ich habe Mitgefühl mit anderen Menschen.

NEIN, nicht mehr als gewöhnlich

JA, sehr viel mehr als gewöhnlich

1. Ich fühle mich mit der Natur verbunden.

NEIN, nicht mehr als gewöhnlich

JA, sehr viel mehr als gewöhnlich

1. Ich bin bereit, mich auf enge Beziehungen einzulassen.

NEIN, nicht mehr als gewöhnlich

JA, sehr viel mehr als gewöhnlich

1. Ich fühle mich kreativ.

NEIN, nicht mehr als gewöhnlich

JA, sehr viel mehr als gewöhnlich

1. Ich nehme Schönheit auch in kleinen Details wahr (z.B. einer menschlichen Stimme, einer Blume, einem Kunstwerk).

NEIN, nicht mehr als gewöhnlich

JA, sehr viel mehr als gewöhnlich

1. Ich nehme die Natur um mich herum wahr.

NEIN, nicht mehr als gewöhnlich

JA, sehr viel mehr als gewöhnlich

1. Mein Denken ist phantasievoll.

NEIN, nicht mehr als gewöhnlich

JA, sehr viel mehr als gewöhnlich

1. Ich fühle, dass alles eins ist.

NEIN, nicht mehr als gewöhnlich

JA, sehr viel mehr als gewöhnlich

**Afterglow Inventory (AGI) Scoring Instructions**

Visual analogue scales (VAS) printed on DIN A4 paper (210 x 297 mm) should be precisely 100 mm long. Item scores range from 0 to 100. Each item score is read out by measuring the horizontal distance between the left endpoint of the VAS and the position marked by the test subject in mm. Scores on the main scale and subscales are calculated as follows.

Subscales:

| **Vitality** | = (Item 01 + Item 02 + Item 07 + Item 08 + Item 11 + Item 12  + Item 14 + Item 16) / 8 |
| --- | --- |
| **Transpersonal Aspects** | = (Item 05 + Item 06 + Item 09 + Item 24) / 4 |
| **Inspiration/Creativity** | = (Item 10 + Item 15 + Item 20 + Item 23) / 4 |
| **Interpersonal Relationships** | = (Item 04 + Item 13 + Item 17 + Item 19) / 4 |
| **Relationship to Nature** | = (Item 03 + Item 18 + Item 21 + Item 22) / 4 |

Main Scale:

| **AGI mean score** | = Sum of all 24 items / 24 |
| --- | --- |
